# VA Peer Providers Supporting Physical Health and Wellness Among Aging Veterans With Mental Health Conditions

**DOI:** 10.1093/geroni/igaa057.2155

**Published:** 2020-12-16

**Authors:** Anjana Muralidharan, Amanda Peeples, Alicia Lucksted, Richard Goldberg

**Affiliations:** 1 VA Capitol Healthcare Network, Baltimore, Maryland, United States; 2 US Department of Veterans Affairs, Baltimore, Maryland, United States; 3 Veterans Affairs Capitol Healthcare Network, Baltimore, Maryland, United States

## Abstract

There is a growing evidence base for the utility of peers in supporting physical health outcomes among aging Veterans with mental illness. This talk will consider two questions: (1) what does it mean to be a “peer” when the focus is improving physical health, and (2) how does peer support promote health behavior change? In considering these questions, select peer-delivered interventions recently or currently being tested in the VA will be discussed. Data from qualitative interviews (N=16; ages 47-75) from a recent RCT of Living Well, a peer co-facilitated group intervention promoting illness self-management, will be presented. These data shed light on the peer role, especially the role of peer self-disclosure in promoting group cohesion, social learning, self-efficacy, and health behavior change. Notably, when physical health is the focus, participants relate to peer providers across diverse characteristics, and not necessarily based on a shared lived experience of mental illness.

